# Maternal size and body condition predict the amount of post‐fertilization maternal provisioning in matrotrophic fish

**DOI:** 10.1002/ece3.4542

**Published:** 2018-12-11

**Authors:** Andres Hagmayer, Andrew I. Furness, David N. Reznick, Bart J. A. Pollux

**Affiliations:** ^1^ Department of Animal Sciences Wageningen University Wageningen The Netherlands; ^2^ Department of Ecology and Evolutionary Biology University of California Irvine California; ^3^ School of Environmental Sciences University of Hull Hull UK; ^4^ Department of Biology University of California Riverside California

**Keywords:** live‐bearing, maternal effect, matrotrophy, placenta, placentotrophy, Poeciliidae, superfetation, viviparity

## Abstract

Maternal effects often provide a mechanism for adaptive transgenerational phenotypic plasticity. The maternal phenotype can profoundly influence the potential for such environmentally induced adjustments of the offspring phenotype, causing correlations between offspring and maternal traits. Here, we study potential effects of the maternal phenotype on offspring provisioning prior to and during gestation in the matrotrophic live‐bearing fish species *Poeciliopsis retropinna*. Specifically, we examine how maternal traits such as body fat, lean mass, and length relate to pre‐ (i.e., allocation to the egg prior to fertilization) and post‐fertilization (i.e., allocation to the embryo during pregnancy) maternal provisioning and how this ultimately affects offspring size and body composition at birth. We show that pre‐ and post‐fertilization maternal provisioning is associated with maternal length and body fat, but not with maternal lean mass. Maternal length is proportionally associated with egg mass at fertilization and offspring mass at birth, notably without changing the ratio of pre‐ to post‐fertilization maternal provisioning. This ratio, referred to as the matrotrophy index (MI), is often used to quantify the level of matrotrophy. By contrast, the proportion of maternal body fat is positively associated with post‐fertilization, but not pre‐fertilization, maternal provisioning and consequently is strongly positively correlated with the MI. We furthermore found that the composition of embryos changes throughout pregnancy. Females invest first in embryo lean mass, and then allocate fat reserves to embryos very late in pregnancy. We argue that this delay in fat allocation may be adaptive, because it delays an unnecessary high reproductive burden to the mother during earlier stages of pregnancy, potentially leading to a more slender body shape and improved locomotor performance. In conclusion, our study suggests that (a) offspring size at birth is a plastic trait that is predicted by both maternal length and body fat, and (b) the MI is a plastic trait that is predicted solely by the proportion of maternal body fat. It herewith provides new insights into the potential maternal causes and consequences of embryo provisioning during pregnancy in matrotrophic live‐bearing species.

## 1 INTRODUCTION

Maternal effects represent the influence of the mother's phenotype on the offspring phenotype independently of the female's genetic contribution to her offspring (Mousseau & Fox, 1998a). In species where a female's environment is a reliable predictor of the environmental conditions that her future offspring are likely to experience, females may evolve the ability to adjust offspring phenotype in ways that best prepare them for life in their future environment. Here, maternal effects will provide a mechanism for adaptive transgenerational phenotypic plasticity (Mousseau & Fox, 1998b).

Maternal effects often entail complex, multiple interacting trade‐offs. Maternal nutrient provisioning to offspring, for instance, can have profound implications for offspring size at birth and hence offspring survival (Mousseau & Fox, 1998a). By changing the offspring phenotype, maternal provisioning may also directly affect maternal fitness, as offspring size is commonly constrained by offspring number (Stearns, 1992). The optimal offspring size is given by the offspring size‐performance relationship that is determined by the environment experienced by the offspring (Fox & Czesak, 2000; Kaplan, 1992; Marshall & Keough, 2008a). Thus, selection is expected to favor the production of differently sized offspring in different environments (Mousseau & Fox, 1998b). Generally, selection favors the production of larger offspring in relatively harsher environments (Marshall, Heppell, Munch, & Warner, 2010; Sibly & Calow, 1983). For instance, strong intra‐ and interspecific competition typically favors larger offspring (Parker & Begon, 1986), as larger offspring have been shown to be better competitors for food (Bashey, 2008; Leips, Rodd, & Travis, 2013). In addition, a variety of other environmental factors, including positive size‐dependent predation, low temperature, salinity, or food availability can induce adaptive plasticity leading to an increase in offspring size (Jørgensen, Auer, & Reznick, 2011; Kaplan, 1992; Marshall & Keough, 2008b).

It has also been argued that the maternal phenotype can significantly influence the potential for environmentally induced adjustments of the offspring phenotype (Marshall & Keough, 2008b; Mousseau & Fox, 1998b). This causes correlations between offspring size and maternal traits such as body condition, size, and age across a wide range of taxa (Stearns, 1992). In general, better‐conditioned, larger and older mothers are often seen to produce larger offspring (Berkeley, Chapman, & Sogard, 2004; Marshall & Keough, 2008b; Marshall et al., 2010; Roff, 1992). Moreover, large mothers typically produce more offspring than small mothers (Calder, 1984). A high rate of offspring production may lead to density‐dependent sibling competition. Under this condition, mothers gain fitness benefits by producing larger, competitive offspring (Leips et al., 2013; Parker & Begon, 1986). In live‐bearers, the survival of the offspring to birth depends on the survival of the mother. If the mother's survival increases with her body size, a theoretical model also predicts a positive mother–offspring size relationship (Jørgensen et al., 2011). Alternatively, size or age‐related differential maternal provisioning might be driven by morphological and physiological constraints that limit the maximal offspring size that a female can produce, rather than being caused by response to selection (Congdon & Gibbons, 1987; Fox & Czesak, 2000). Thus, maternal phenotype can have important effects, either directly or indirectly, on maternal provisioning, making condition‐, size‐ or age‐related differential maternal provisioning an important potential cause of variation in offspring size and fitness.

Although mother–offspring size relationships have been shown to be common in a wide range of taxa (Stearns, 1992), little is known about the influence of maternal condition, size, and age on embryo provisioning in matrotrophic live‐bearing species. Instead of allocating all resources to the eggs prior to fertilization (i.e., lecithotrophy), matrotrophic species transfer their nutrients to the developing embryos throughout gestation (Wourms, 1981). Since they continuously supply their developing embryos with resources (Pollux, Pires, Banet, & Reznick, 2009; Wourms, 1981), the timing of determining brood size and offspring size can be decoupled (Pollux & Reznick, 2011; Reznick, Callahan, & Llauredo, 1996). Whereas brood size is determined prior to fertilization based on prior food availability, offspring size is determined after fertilization based on food availability throughout gestation (Pollux & Reznick, 2011; Reznick, Callahan, et al., 1996). Matrotrophy, therefore, has been suggested to be a maladaptive strategy in environments where food is scarce or fluctuating because low food availability causes the production of smaller offspring at a time when being large at birth is favored (Pollux & Reznick, 2011; Reznick, Callahan, et al., 1996; Trexler & DeAngelis, 2003, 2010). It has further been argued that matrotrophic species would be better buffered against fluctuating food availability, if they had the ability to diminish brood size via abortion and resorb the invested energy and/or the ability to store large quantities of fat reserves that prevent females from undernourishing their embryos (Trexler, 1997; Trexler & DeAngelis, 2003). Although there is no evidence for embryo abortion due to low food availability in matrotrophic species of the family Poeciliidae, they do sacrifice their fat reserves to sustain their developing embryos under such conditions (Banet, Au, & Reznick, 2010; Banet & Reznick, 2008; Pollux & Reznick, 2011; Reznick, Callahan, et al., 1996). Therefore, maternal size and fat reserves are of evolutionary relevance, especially under adverse food conditions.

**Figure 1:**
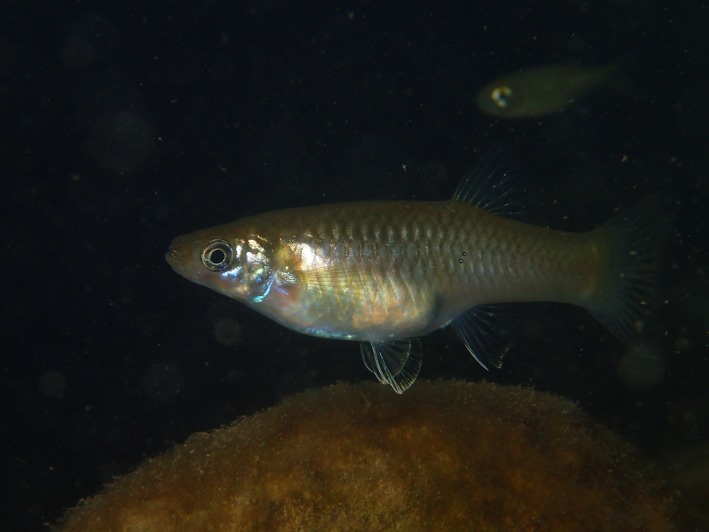
Female *Poeciliopsis retropinna* (family Poeciliidae). Photograph: Andrew Furness

Here, we study potential maternal causes and consequences of embryo provisioning during gestation in the matrotrophic live‐bearing fish species *Poeciliopsis retropinna* (family Poeciliidae; Regan, 1908; Figure 1). Specifically, we quantify the relationship of maternal traits (i.e., the proportion of maternal body fat, lean mass, and standard length) with: (a) embryo size and body composition during pregnancy, (b) offspring size and body composition at birth, (c) the matrotrophy index (MI), which is a measure of post‐fertilization maternal provisioning (Pollux, Meredith, Springer, Garland, & Reznick, 2014; Reznick, Mateos, & Springer, 2002), and (d) fecundity. Body fat is believed to be a good indicator of fish condition (Leips et al., 2013) and standard length has been shown to be a good proxy for age (Reznick, Butler, Rodd, & Ross, 1996). *Poeciliopsis retropinna* has superfetation, or the ability to carry several broods at various developmental stages (Turner, 1937), making it a good study system to obtain reliable estimates of embryo mass throughout gestation. We first quantify phenotypic variation in embryo traits (i.e., dry mass, body fat, and lean mass) as a function of developmental stage (Haynes, 1995). The number of embryos is used here as a surrogate measure for maternal fecundity. We then correlate this variation to maternal traits (i.e., the proportion of maternal body fat, lean mass, and standard length) to detect maternal condition‐, size‐ and/or age‐related offspring provisioning. Furthermore, we evaluate the consequences for the MI and fecundity. Finally, we discuss our results in light of the evolution of matrotrophy in live‐bearing animals.

## 2 METHODS

### 2.1 Study species and collection sites

*Poeciliopsis retropinna*, a live‐bearing fish species in the family Poeciliidae, reaches a maximum standard length of approximately 80 mm. This species is found in freshwater streams of all current velocities from sea level to 940 m elevation in Costa Rica and Panama (Bussing, 2002). During gestation, *P. retropinna* females transfer nutrients to developing embryos via a placenta‐like structure (i.e., they exhibit matrotrophy) (Pollux et al., 2009). The degree of post‐fertilization maternal provisioning in this species is extensive, with offspring increasing in dry mass more than 100‐fold during gestation (MI = 117; Reznick et al., 2002).

During March 2013, *P. retropinna* were collected at five different locations in the Rio Terraba drainage in the province of Puntarenas, Costa Rica (Supporting Information Table S1). At each location, 10–30 adult females were collected using seine and cast nets, euthanized with an overdose of MS‐222 (Sigma‐Aldrich Co., St. Louis, Missouri, USA), and preserved in 5% formaldehyde (Fisher Scientific, Fair Lawn, New Jersey, USA). The fish samples were transported to the Reznick laboratory (University of California Riverside, USA) for anatomical dissections.

### 2.2 Laboratory measurements

The standard length of preserved specimens was measured to the nearest mm from the tip of the upper jaw to the outer margin of the hypural plate, using a caliper. Female dry mass was measured to the nearest 0.01 mg on a Mettler Toledo AE163 Microbalance (Mettler Instruments Corp., Hightstown, New Jersey, USA) after removing the ovary and air‐drying the female overnight at 60°C in a drying oven. Only four of the five sampled populations contained pregnant females (Supporting Information Table S1); therefore, all subsequent anatomical and statistical analyses were carried out only with the females from these four populations. Female lean mass was measured by extracting the fat twice with anhydrous diethyl ether (Fisher Scientific) to remove triglycerides, and by subsequently air‐drying and re‐weighting the female (see above). The proportion of maternal body fat was then calculated by subtracting maternal lean mass from maternal dry mass divided by maternal dry mass. The ovaries were dissected to count the number of embryos (i.e., fecundity), and to determine the developmental stage and average mass of the embryos for a given brood. Since the embryos are counted across all broods, fecundity reflects a combination between the effects of brood size and superfetation. The developmental stages are based on morphological criteria described in Haynes (1995) and range from 0 (eggs at fertilization, no development) to 45 (fully developed embryos), with stage 50 representing newborn offspring (Haynes, 1995; Reznick et al., 2002). The embryo dry mass for a given brood was calculated as the dry mass of the brood, measured to the nearest 0.01 mg after air‐drying overnight at 60°C (see above), divided by the number of embryos in the brood (Pollux & Reznick, 2011). The embryo lean mass was then measured by extracting the fat twice with anhydrous diethyl ether then air‐drying and re‐weighting the brood, and by dividing by the number of embryos. The proportion of embryo body fat was calculated as described above. Fecundity was calculated by excluding stage 0 embryos (since it was difficult to assess if they were all fertilized). A detailed explanation of the measured maternal and embryo traits is given in Table 1.

**Table 1:** Summary of maternal and embryo traits

Maternal traits	
Standard length	Length from the tip of the upper jaw to the outer margin of the hypural plate; used as a proxy for age
Dry mass	Dry mass after removing ovary
Lean mass	Dry mass after removing ovary and triglycerides
Body fat	Maternal lean mass subtracted from maternal dry mass
Proportion body fat	Maternal body fat divided by maternal dry mass; used as a proxy for body condition
Fecundity	Number of embryos carried by a female counted across all broods
Embryo traits	
Dry mass	Brood dry mass divided by the number of embryos in the brood
Lean mass	Brood dry mass after removing triglycerides divided by the number of embryos in the brood
Body fat	Embryo lean mass subtracted from embryo dry mass
Proportion body fat	Embryo body fat divided by embryo dry mass; used as a proxy for body condition
Developmental stage	Based on morphological criteria that range from 0 (eggs at fertilization, no development) to 45 (fully developed embryos), with stage 50 representing newborn offspring

### 2.3 Statistical analysis

To identify potential condition‐, size‐ and/or age‐related effects on embryo provisioning, a series of linear mixed effect models were fitted by Maximum Likelihood. The best model was selected on the basis of Akaike's information criterion adjusted for small sample sizes (AICc) (Burnham & Anderson, 2002). Ln‐transformed embryo dry mass was the response variable. Fixed effects in the full model included the proportion of maternal body fat, lean mass, standard length and the interaction between each of these variables and the developmental stage of the embryos. Mother identity was fitted as random intercept to correct for pseudo‐replication and for between‐female variation in maternal provisioning that is not accounted by the fixed effects. Population identity was fitted as an additional random intercept accounting for spatial non‐independence of observations.

A significant interaction effect between the proportion of maternal body fat, lean mass, or standard length and embryo developmental stage implies that the maternal effect is not constant during pregnancy and depends on the developmental stage of the embryos. Consequently, a change in the maternal trait will lead to a change in the matrotrophy index (MI), defined as the estimated dry mass of the offspring at birth (stage 45; derived from the model equation) divided by the estimated dry mass of eggs at fertilization (stage 0), (Pollux et al., 2014; Reznick et al., 2002). A non‐significant interaction effect, by contrast, implies that the maternal trait is proportionally associated with egg mass at fertilization and offspring mass at birth without changing the MI. In case of a significant interaction term, the relationship between the corresponding maternal trait and egg mass at fertilization is given by the maternal main effect, since main effects are estimated where all other predictors are zero (i.e., stage 0; eggs at fertilization) (Schielzeth, 2010). The relationship between the maternal trait and offspring mass at birth, by contrast, is given by the maternal main effect after subtracting the developmental stage at birth (*s* *_birth_*) from the actual developmental stage of the *j*th brood in the *i*th mother (*s*_*i,j*_) in a second model (i.e., *s*_*i,j*_ * = *s*_*i,j*_ *−s*_*birth*_).

To illustrate the association of the maternal traits with embryo body composition (i.e., body fat and lean mass) during gestation, we fit embryo body fat, embryo lean mass, and the proportion of embryo body fat as a function of the same fixed effect structure found to best explain embryo dry mass according to the AICc. Consequently, all the model structures are uniform, which facilitates parameter comparisons and interpretations. Mother and population identity were fitted as random intercepts to correct for pseudo‐replication, between‐female and ‐population variation in maternal provisioning that is not accounted by the fixed effects. In case of a significant interaction term, the relationship between the corresponding maternal trait and offspring mass at birth is again quantified by subtracting the developmental stage at birth from the actual developmental stage of a given brood.

We tested the association of the maternal traits with fecundity by fitting a generalized linear mixed effect model by Maximum Likelihood and a log link for the Poisson‐distributed response. The fixed effects included the maternal traits found to predict embryo dry mass. Population identity was included as a random effect as was the latest developmental stage of embryos (to account for females early in the reproductive cycle).

To optimize normality and homoscedasticity of the model residuals, absolute embryo weights (i.e., dry mass, lean mass, and body fat) were ln‐transformed, the proportion of maternal and embryo body fat was arcsin square‐root transformed, and developmental stage was transformed to the square‐root of its third power. To compare the strength of the relationship with individual maternal traits, the regression coefficients (*β*) were additionally standardized by multiplying with the phenotypic standard deviation of the maternal trait and by dividing by the phenotypic standard deviation of the response variable (*β*′) (Schielzeth, 2010). All the analyses were carried out in R v 3.1.3 (R Core Team, 2015): Mixed models were fitted using the lme4 package (Bates, Mächler, Bolker, & Walker, 2015), and significance tests for the fixed effects were performed with lmerTest (Kuznetsova, Brockhoff, & Christensen, 2016).

## 3 RESULTS

### 3.1 Maternal effects on embryo dry mass

**Figure 2:**
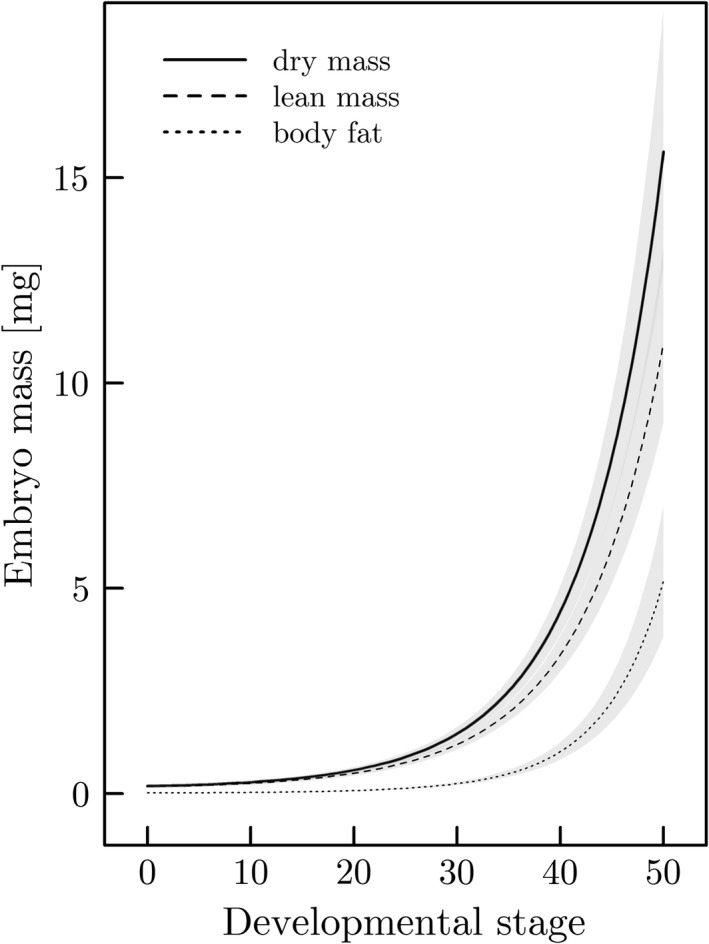
Embryo dry mass, lean mass and body fat as a function of developmental stage of embryos (±95% CI) predicted for a female of average standard length (i.e., ${\overset{¯}{y}}_{\mathrm{standard}\;\mathrm{length}}$ = 67 mm) and average proportion of maternal body fat (i.e., ${\overset{¯}{y}}_{\mathrm{body}\;\mathrm{fat}}$ = 0.20). This prediction is based on the model parameters described in Supporting Information Tables S3–S5

**Figure 3:**
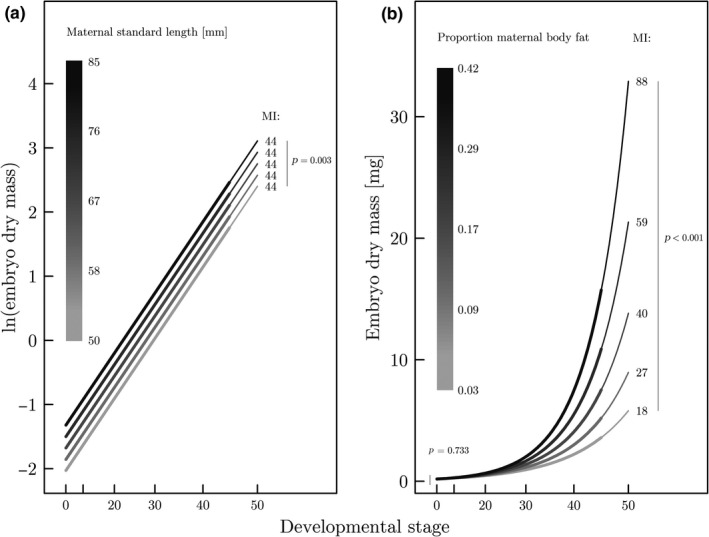
(a) The relationship between maternal standard length and embryo dry mass throughout gestation. Embryo dry mass is predicted as a function of developmental stage, for the population mean of the proportion of maternal body fat (i.e., ${\overset{¯}{y}}_{\mathrm{body}\;\mathrm{fat}}$ = 0.20), and for five hypothetical females of different length (i.e., 50, 58, 67, 76, and 85 mm). The prediction is based on the model that best describes variation in embryo dry mass (*n *=* *68 broods from 40 mothers; Supporting Information Tables S2 and S3). To illustrate the proportional association of maternal standard length with egg dry mass at fertilization and offspring dry mass at birth, both axes are shown on the same scale as used to fit the model (i.e., *y*‐axis: ln‐scale, *x*‐axis: $\sqrt{x^{3}}$). Please note that the *y*‐intercepts on ln‐scale are significantly different (*t*_30.025_ = 3.214, *p *=* *0.003), while the slopes are the same. This suggests that female length is proportionally associated with egg mass at fertilization and offspring mass at birth, which means that the predicted matrotrophy indices (shown on the right) remain unaffected (i.e., MI = 44 for all five females). The thin‐lined parts correspond to the time of birth (i.e., developmental stage 45–50). (b) The interaction effect between the proportion of maternal body fat and developmental stage of embryos estimated in the best model explaining variation in embryo dry mass (*n *=* *68 broods from 40 mothers; Supporting Information Table S2 and S3). Embryo dry mass is predicted as a function of developmental stage, for the population mean of maternal standard length (i.e., ${\overset{¯}{y}}_{\mathrm{standard}\;\mathrm{length}}$ = 67 mm), and for five hypothetical females with different proportions of body fat (i.e., 0.03, 0.09, 0.17, 0.29, and 0.42 proportion body fat). The *x*‐axis is shown on the same scale as used to fit the model (i.e. $\sqrt{x^{3}}$). The *y*‐axis is shown on the untransformed scale to illustrate the exponential increase in embryo dry mass. Please note that the y‐intercepts are not significantly different (*t*_54.291_ = −0.343, *p *=* *0.733), while the slopes differ significantly among the five females (*t*_64.077_ = 3.723, *p *<* *0.001; Supporting Information Table S3). This indicates that maternal body fat is not related to the amount of pre‐fertilization maternal provisioning, but strongly correlates with the amount of post‐fertilization provisioning to the embryo during pregnancy and hence offspring dry mass at birth and the MI (*t*_74.654_ = 5.369, *p *<* *0.001)

Variation in embryo dry mass throughout development is best explained by the proportion of maternal body fat, standard length, developmental stage of the embryo and an interaction between the proportion of maternal body fat and the developmental stage of the embryo (Supporting Information Tables S2 and S3). Maternal lean mass and the interaction between both maternal lean mass and standard length with developmental stage were excluded as predictors in the final model (Supporting Information Table S2). Embryo dry mass (mg) significantly increases throughout development, especially during late developmental stages (*t*_59.442_ = 5.638, *p *<* *0.001; Supporting Information Table S3; Figure 2). An increase in maternal standard length (mm) is associated with a proportional increase in both egg dry mass (mg) at fertilization and offspring dry mass (mg) at birth (*t*_30.025_ = 3.214, *p *=* *0.003; Supporting Information Table S3), and, hence, the MI does not change (Figure 3a). By contrast, an increase in the proportion of maternal body fat is associated with a significant increase in offspring dry mass (mg) at birth (*t*_74.654_ **=* *5.369, *p *<* *0.001; Supporting Information Table S3.b), but not with egg dry mass (mg) at fertilization (*t*_54.291_ **=* *−0.343, *p *=* *0.733; Supporting Information Table S3.a). An increase in the proportion of maternal body fat thus predicts a significantly higher MI (Figure 3b).

### 3.2 Maternal effects on embryo lean mass

Embryo lean mass (mg) significantly increases throughout development, especially late in pregnancy (*t*_59.460_ **=* *5.776, *p *<* *0.001; Supporting Information Table S4; Figure 2). In addition, embryo lean mass increased as a function of increased maternal body fat and standard length (Supporting Information Table S4). Maternal standard length (mm) is proportionally related to egg lean mass at fertilization and offspring lean mass at birth (*t*_30.608_ **=* *3.379, *p *=* *0.002; Supporting Information Table S4). The proportion of maternal body fat is positively associated with offspring lean mass (mg) at birth (*t*_73.671_ **=* *5.046, *p *<* *0.001; Supporting Information Table S4.b), but is not significantly correlated to egg lean mass (mg) at fertilization (*t*_54.965_ **=* *0.066, *p *=* *0.948; Supporting Information Table S4.a).

### 3.3 Maternal effects on absolute amount of embryo body fat

Embryo body fat (mg) significantly increases during development, but only very late in pregnancy (*t*_56.922_ **=* *5.499, *p *<* *0.001; Supporting Information Table S5; Figure 2). Maternal standard length (mm) is not significantly related to the amount of embryo body fat (mg) (*t*_32.052_ **=* *0.591, *p *=* *0.559; Supporting Information Table S5). The proportion of maternal body fat, on the other hand, is significantly associated with the amount of body fat (mg) in the offspring at birth (*t*_73.273_ **=* *4.373, *p *<* *0.001; Supporting Information Table S5.b), yet does not correlate with the quantity of fat (mg) in the egg at fertilization (*t*_56.605_ **=* *0.551, *p *=* *0.584; Supporting Information Table S5.a).

### 3.4 Maternal effects on proportion of embryo body fat

**Figure 4:**
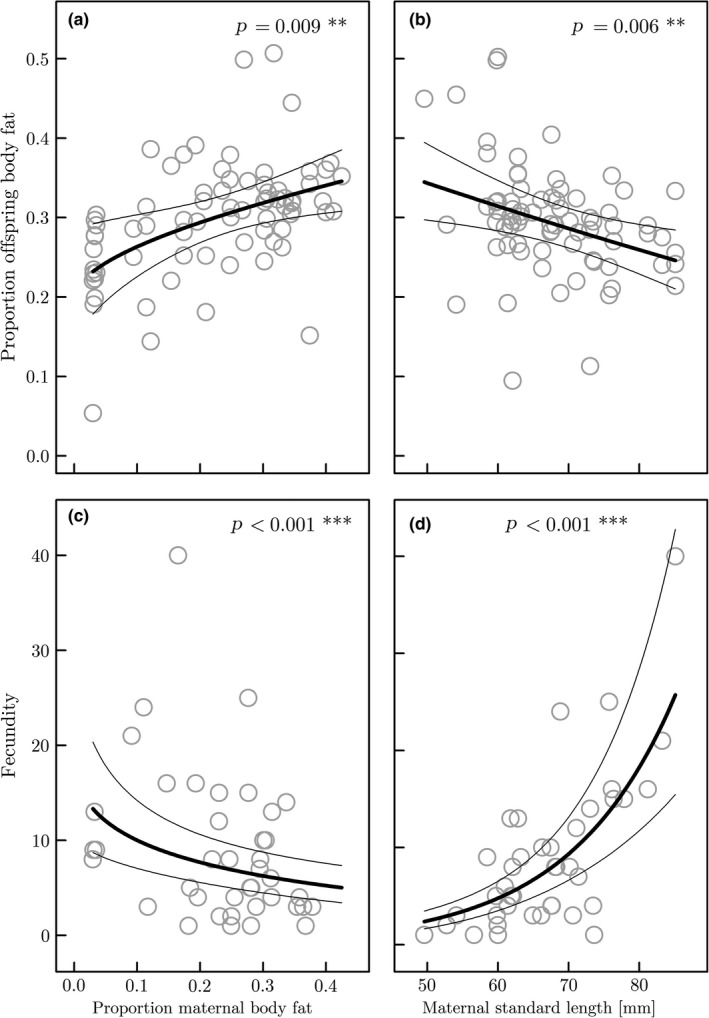
The top panel shows the association of (a) the proportion of maternal body fat and (b) maternal standard length with the proportion of offspring body fat at birth (±95% CI) estimated in the model described in Supporting Information Table S6.b. Residuals and model fit account for maternal standard length in (a) and the proportion of maternal body fat in (b) that are kept constant at their population mean (i.e., ${\overset{¯}{y}}_{\mathrm{standard}\;\mathrm{length}}$ = 67 mm, ${\overset{¯}{y}}_{\mathrm{body}\;\mathrm{fat}}$ = 0.20). The bottom panel shows the association of the number of embryos (i.e., fecundity) with (c) proportion of maternal body fat, and (d) maternal standard length (±95% CI) estimated in the model described in Supporting Information Table S7. Model fit accounts for maternal standard length in (c) and the proportion of maternal body fat in (d) that are kept constant at their population mean (i.e., ${\overset{¯}{y}}_{\mathrm{standard}\;\mathrm{length}}$ = 67 mm, ${\overset{¯}{y}}_{\mathrm{body}\;\mathrm{fat}}$ = 0.20)

The proportion of embryo body fat also increases significantly throughout gestation (*t*_74_ **=* *2.084, *p *=* *0.041; Supporting Information Table S6). Maternal standard length is negatively related to the proportion of embryo body fat throughout development (*t*_74_ **=* *−2.849, *p *=* *0.006; Supporting Information Table S6; Figure 4b). The proportion of maternal body fat is positively associated with the proportion of offspring body fat at birth (*t*_74_ **=* *2.675, *p *=* *0.009; Supporting Information Table S6.b; Figure 4a), but does not correlate with the proportion of egg fat at fertilization (*t*_74_ **=* *−0.109, *p *=* *0.914; Supporting Information Table S6.a).

### 3.5 Fecundity

Both the proportion of maternal body fat and standard length were significantly associated with maternal fecundity (Supporting Information Table S7). Maternal fecundity is negatively related to an increasing proportion of maternal body fat (*z *=* *−4.022, *p *<* *0.001; Supporting Information Table S7; Figure 4c) and strongly positively related to an increasing standard length (*z *=* *7.606, *p *<* *0.001; Supporting Information Table S7; Figure 4d).

## 4 DISCUSSION

Maternal traits can profoundly influence the development and hence phenotype of offspring at birth (Mousseau & Fox, 1998a,1998b). We studied the associations of maternal traits with offspring provisioning prior to and during gestation in field‐collected specimens of the matrotrophic live‐bearing fish species *Poeciliopsis retropinna* (family Poeciliidae). We found temporal variation in the composition of embryos throughout gestation: While lean mass is continuously accumulating, fat reserves are predominantly allocated to embryos very late in pregnancy (Figure 2). Furthermore, we found that the level of maternal provisioning to the developing embryos correlates with the proportion of maternal body fat and standard length, but not with maternal lean mass. The proportion of maternal body fat is positively associated with offspring dry mass at birth, but not with egg dry mass at fertilization. This suggests that an increase in the proportion of maternal body fat is likely associated with an increase in the matrotrophy index (MI). By contrast, maternal standard length is proportionally associated with both egg dry mass at fertilization and offspring dry mass at birth, indicating that the MI does not change with increasing maternal standard length (Figure 3a,b).

### 4.1 Effect of maternal body fat

We found that the proportion of maternal body fat in *P. retropinna* is associated with an increase in post‐ but not pre‐fertilization maternal provisioning. Females that have more fat reserves produce larger offspring at birth than females with lower fat reserves, without investing more in egg size at fertilization. Consequently, females with more fat reserves are likely to have higher “levels of matrotrophy,” or in other words, higher MIs. The potential mechanisms behind the increased level of post‐fertilization maternal provisioning by better‐conditioned mothers are unclear. In general, the amount of resources a female can transfer to her developing offspring per unit of time is likely the result of a balance between maternal energy uptake (via feeding), her own caloric utilization (maintenance) and the amount of excess energy that is subsequently available for reproduction (Stearns, 1992). It is possible that better‐conditioned females that carry more fat reserves simply have more energy available that can be used to invest in developing embryos. In this case, rather than being an adaptive strategy, worse‐conditioned females would be physiologically hindered to produce larger offspring.

Better‐conditioned mothers (i.e., those carrying larger fat reserves) produced fewer, but better‐conditioned offspring. One potential explanation for this is the trade‐off between offspring size and number; the production of large and high‐quality offspring may necessarily entail the production of fewer offspring owing to the limited size of the female body cavity (Stearns, 1992). Moreover, variation in offspring size and composition at birth is likely to have profound effects on offspring fitness, since larger offspring size at birth and a higher proportion of offspring body fat both have been shown to improve survival under specific environmental conditions. Larger offspring, for instance, perform better when food is scarce and competition for food is high, though the advantage of being relatively larger disappears when competition is insignificant (Bashey, 2008; Leips et al., 2013; Parker & Begon, 1986). In fish, larger offspring have better escape performance (Dial, Reznick, Brainerd, & Marshall, 2016; Gibb, Swanson, Wesp, Landels, & Liu, 2006), which is likely to increase survival in high predation environments. Similarly, larger offspring are more resistant to starvation (Gliwicz & Guisande, 1992), probably because larger offspring contain more maternal reserves that prevent them from starvation under adverse food conditions (Tessier, Henry, Goulden, & Durand, 1983). In situations where the maternal environment provides a reliable predictor of future environmental conditions that offspring are likely to experience, females may evolve the ability to adaptively adjust offspring phenotype at birth as a response to environmental cues (Mousseau & Fox, 1998b). Matrotrophic fish, for instance, have been shown to increase offspring size in response to increasing conspecific densities (Leips et al., 2013). This pattern is interpreted as adaptive as it increases offspring fitness under highly competitive conditions.

Here, we show that the maternal phenotype is strongly correlated with offspring phenotype in a matrotophic fish; offspring size and composition at birth are strongly associated with maternal body condition. Although we cannot determine whether offspring size and composition at birth is due to adaptive transgenerational plasticity or morphological and/or physiological constraints, the observed pattern of differential embryo provisioning is seemingly more consistent with a physiological constraint. In matrotrophic fish, it has been proposed that maternal fat reserves buffer females against low or fluctuating food availability (Trexler, 1997; Trexler & DeAngelis, 2003). Since matrotrophic species continuously supply their developing embryos with resources (Pollux et al., 2009; Wourms, 1981), the timing of determining brood size and offspring size are likely to be decoupled (Pollux & Reznick, 2011; Reznick, Callahan, et al., 1996). Whereas brood size is determined prior to fertilization based on current food availability, offspring size is determined after fertilization based on food availability throughout gestation (Pollux & Reznick, 2011; Reznick, Callahan, et al., 1996). In environments where food is scarce or fluctuating, therefore, matrotrophic species presumably run the risk of fertilizing more eggs than can be thoroughly provisioned during gestation. Stored maternal fat reserves could theoretically prevent females from undernourishing their embryos and enable them to sustain their brood under adverse food conditions (Trexler, 1997; Trexler & DeAngelis, 2003). However, available evidence suggests that maternal body fat might not fully buffer matrotrophic females against unfavorable environmental conditions (Pollux & Reznick, 2011). The matrotrophic fish species *Heterandria formosa, Poeciliopsis turneri, P. prolifica* and *Phalloptychus januarius* all responded to low food conditions under laboratory conditions by producing smaller offspring at birth with less fat reserves (Banet & Reznick, 2008; Banet et al., 2010; Pollux & Reznick, 2011; Reznick, Callahan, et al., 1996). In concurrence with these laboratory studies, we show, under natural conditions in field‐collected specimens of *Poeciliopsis retropinna*, that decreasing maternal body condition (i.e., proportion body fat) is negatively associated with offspring size and body condition at birth. Since smaller offspring with less fat reserves are expected to have lower fitness under adverse food and hence highly competitive conditions (Bashey, 2008; Leips et al., 2013; Parker & Begon, 1986; Tessier et al., 1983), matrotrophy has been suggested to be a maladaptive strategy in environments characterized by low or fluctuating food availability (Banet et al., 2010; Pollux & Reznick, 2011; Reznick, Callahan, et al., 1996).

### 4.2 Timing of fat allocation to developing embryos

Live‐bearing fish larvae from the family Poeciliidae are super precocial, having functional prey capture abilities at birth (Lankheet, Stoffers, van Leeuwen, & Pollux, 2016). The newborn's prey capturing ability, however, is far from perfect and requires a rapid integrated development of the visuo‐motor system during the first days after birth to optimize prey capture success rate and ensure a sufficient uptake of resources for survival (Lankheet et al., 2016). This is particularly important when offspring are born in low resource environments where prey availability may be sparse. In these environments, offspring may benefit from having a “back pack with fat reserves” that may help them to survive the first days after birth (Chambers, Leggett, & Brown, 1989).

We found that embryos from the matrotrophic fish species *Poeciliopsis retropinna* gain fat only very late in pregnancy. The same pattern has been shown in placental mammals, where fetal fat mobilization also occurs late in pregnancy (Petterson, Slepetis, Ehrhardt, Dunshea, & Bell, 1994). In mammals, fetal fat deposition during pregnancy is however relatively insignificant (Elphick, Hull, & Broughton Pipkin, 1979), since most lipids are allocated to offspring postnatally, that is, during lactation (Bell, 1995). One might argue that the late (in fish) or relatively insignificant (in mammals) allocation of fat during pregnancy could be an adaptive feature of mobile matrotrophic live‐bearing animals in general, because studies have shown that an increase in reproductive allocation (i.e., the proportion of the mother's mass allocated to developing offspring) during pregnancy can lead to a less slender body shape (Fleuren, Quicazan‐Rubio, van Leeuwen, & Pollux, 2018) and negatively affect female locomotor performance in a wide range of viviparous taxa (e.g., fish, Plaut, 2002; Reznick, Bryant, Roff, Ghalambor, & Chalambor, 2004; reptiles, Seigel, Huggins, & Ford, 1987; and mammals, Noren, Redfern, & Edwards, 2011), which consequently may reduce survival probability (Laidlaw, Condon, & Belk, 2014; Plath, Riesch, Culumber, Streit, & Tobler, 2011). Thus, we suggest that the late allocation of fat might be adaptive, because earlier allocation of resources would unnecessarily increase the reproductive burden suffered by a pregnant female, and negatively impact her locomotor performance and, hence, her chance of survival.

### 4.3 Effect of maternal size

Larger (i.e., longer) females had greater fecundity. This finding is consistent with patterns observed in a wide range of taxa that exhibit indeterminate growth (Reznick, 1983), since fecundity increases as a consequence of space available in the female's body cavity (Shine, 1992). In addition, we found that maternal standard length is positively correlated with offspring size at birth; although this effect is approximately three times smaller than the effect of the proportion of maternal body fat (see standardized regression coefficient *β*′ in Supporting Information Table S3.b). Larger females produced more and larger offspring at birth. Optimality models predict that the total reproductive effort will increase with age, as expected future reproductive success decreases (Pianka & Parker, 1975). Since standard length is positively correlated with age (Reznick, Butler, et al., 1996), the production of more and larger offspring by larger females might be explained by an increased reproductive investment with age, rather than by female size per se.

We further found that maternal length is negatively related with offspring body condition. Larger females produce more and larger offspring, but they contain less fat reserves. As a female's physiology changes during aging, offspring quality might be expected to decrease with maternal age as a consequence of senescence. Offspring of old females have been shown to have reduced survival probability (Descamps, Boutin, Berteaux, & Gaillard, 2008), lower egg hatching success (Kern, Ackermann, Stearns, & Kawecki, 2001), and lower fat reserves (McIntyre & Gooding, 2000). Although the total reproductive investment (i.e., size and number of offspring) is likely to increase with maternal age as a consequence of decreasing future reproductive success (Pianka & Parker, 1975), older mothers might provide their offspring with less fat reserves as a consequence of senescence. Since we do not have direct measurements of a female's age, however, we cannot disentangle maternal size effects due to morphological constraints (i.e., body cavity volume) and maternal age effects due to senescence and/or age‐dependent reproductive effort.

### 4.4 The matrotrophy index is (at least partly) a phenotypically plastic trait

Our results strongly suggest that the MI exhibits phenotypic plasticity and can change throughout a female's lifetime (i.e., due to maternal body condition but not length) or across environmental conditions (e.g., due to food availability). The extent of post‐fertilization maternal provisioning has already been shown to plastically respond to environmental conditions such as food availability in other matrotrophic poeciliids (Banet & Reznick, 2008; Banet et al., 2010; Pollux & Reznick, 2011; Reznick, Callahan, et al., 1996). Specifically, females produced smaller offspring at birth under low food availability. This is likely to reduce the MI. However, in these studies, measurements of the egg phenotype (i.e., the extent of pre‐fertilization maternal provisioning) were either not reported or analyzed, and consequently, it was not possible to make inferences about the MI. Nevertheless, these studies show an almost instantaneous link between food availability and offspring size at birth. Here, we demonstrate that offspring size at birth strongly correlates with maternal body condition and that maternal body condition relates to the MI primarily through post‐, rather than pre‐fertilization maternal provisioning. The extent to which maternal provisioning is a direct consequence of food availability (i.e., nutrients that are provided without transit through maternal fat reserves) and an indirect consequence mediated through maternal body condition (i.e., nutrients that are mobilized from maternal fat reserves) remains to be tested. Whereas under nutrient‐rich conditions females may provision their embryos with resources that are not previously stored as maternal fat, they may increasingly rely on fat reserves when food is scarce, making body condition an important and direct embryonal food source under adverse conditions.

## CONFLICT OF INTEREST

None declared.

## AUTHORS’ CONTRIBUTION

BJAP and AIF conceived the ideas and planned the fieldwork; BJAP and AIF collected the data; AIF carried out the dissections; AH analyzed the data, wrote the first draft of the manuscript and finalized the manuscript with comments from AIF, DNR and BJAP.

## DATA ACCESSIBILITY

Data available from the Dryad Digital Repository: https://doi.org/10.5061/dryad.qt8744c.
