# Supplementary material for: Maternal size and body condition predict the amount of post‐fertilization maternal provisioning in matrotrophic fish
Source: Ecol Evol. 2018 Dec 11;8(24):12386–96. doi: 10.1002/ece3.4542 (PMC6308890; doi:10.1002/ece3.4542)
Supplement: Supplementary file 1 [file ECE3-8-12386-s001.pdf]

Table S1. Sampling locations (plus coordinates) in the Rio Terraba (Costa Rica), sampling dates and number of collected (*n*) and pregnant (n_{preg}) females at each location.

Location	Coordinates	Sampling date	*n*	n_{preg}
Pacuar	N 9° 21', W 83° 44 '	25.03.2013	20	3
Pedregoso	N 9° 21 ', W 83° 43'	25.03.2013	30	0
Ceibo	N 9° 9 ', W 83° 23'	26.03.2013	24	8
Sucio	N 8° 49', W 82° 55'	27.03.2013	24	23
Copal	N 8° 48', W 82° 55'	27.03.2013	10	6
