# Supplementary material for: Maternal size and body condition predict the amount of post‐fertilization maternal provisioning in matrotrophic fish
Source: Ecol Evol. 2018 Dec 11;8(24):12386–96. doi: 10.1002/ece3.4542 (PMC6308890; doi:10.1002/ece3.4542)
Supplement: Supplementary file 2 [file ECE3-8-12386-s002.pdf]

Table S2. Parameter estimates of the linear mixed effect models predicting variation in ln-transformed embryo dry mass with ΔAICc≤10. Parameter interpretation is based on the best model according to the AICc (i.e. model 1).

Model	Int	s	BF	LM	SL	BF×s	LM×s	SL×s	*df*	logLik	AICc	ΔAICc	weight
1	-1.825	0.008	-0.187	NA	0.204	0.011	NA	NA	8	-42.464	103.111	0	0.241
2	-1.221	0.008	-0.369	-0.76	0.573	0.011	NA	NA	9	-41.189	103.146	0.036	0.236
3	-1.192	0.008	-1.142	NA	NA	0.01	NA	NA	7	-44.145	103.962	0.851	0.157
4	-1.065	0.007	-0.36	-0.915	0.599	0.011	0.001	NA	10	-40.955	105.347	2.237	0.079
5	-0.295	0.002	-0.564	-1.96	1.133	0.012	0.007	-0.004	11	-39.732	105.654	2.543	0.067
6	-1.82	0.008	-0.182	NA	0.197	0.011	NA	0	9	-42.461	105.692	2.581	0.066
7	-1.187	0.007	-0.356	-0.781	0.56	0.011	NA	0	10	-41.14	105.718	2.608	0.065
8	-2.083	0.008	-0.062	0.346	NA	0.01	NA	NA	8	-43.91	106.002	2.891	0.057
9	-2.032	0.008	-0.05	0.3	NA	0.01	0	NA	9	-43.868	108.506	5.395	0.016
10	-1.682	0.012	NA	NA	NA	NA	NA	NA	5	-50.211	111.292	8.181	0.004
11	-1.915	0.012	0.543	NA	NA	NA	NA	NA	6	-49.757	112.748	9.637	0.002

Int: intercept; s: developmental stage of embryos transformed to the square-root of its third power; BF: arcsin square-root transformed proportion of maternal body fat; LM: maternal lean mass; SL: z-standardized maternal standard length (zero-mean and unit standard deviation); AICc: Akaike's information criterion adjusted for small sample sizes
