# Supplementary material for: Maternal size and body condition predict the amount of post‐fertilization maternal provisioning in matrotrophic fish
Source: Ecol Evol. 2018 Dec 11;8(24):12386–96. doi: 10.1002/ece3.4542 (PMC6308890; doi:10.1002/ece3.4542)
Supplement: Supplementary file 3 [file ECE3-8-12386-s003.pdf]

Table S3.a. Fixed and random effects in explaining variation in ln-transformed embryo dry mass in the best linear mixed effect model according to the AICc. The maternal main effects (i.e. body fat and standard length) quantify the relationship between the corresponding maternal trait and egg dry mass at fertilization. Significance tests for the fixed effects were performed with `lmerTest` (Kuznetsova, Brockhoff & Christensen 2016), and confidence intervals for random effects were calculated using `confint.merMod` function implemented in the R package `lme4` (Bates *et al.* 2015).

Fixed effects							
	β	β'	SE	*df*	*t*	*P*	Sig.
Intercept	-2.928	-	0.441	26.950	-6.633	<0.001	***
developmental stage[a]	0.008	-	0.001	59.442	5.638	<0.001	***
body fat[b]	-0.187	-0.017	0.545	54.291	-0.343	0.733	n.s.
standard length	0.020	0.098	0.006	30.025	3.214	0.003	**
body fat[b] × developmental stage[a]	0.011	-	0.003	64.077	3.723	<0.001	***

Random effects			
	Variance	2.5 % confidence level	97.5 % confidence level
mother identity	0.0034	0.0000	0.0727
population identity	0.0000	0.0000	0.1634
residual	0.1783	0.1174	0.2546

[a] developmental stage transformed to the square-root of its third power; [b] arcsin square-root transformed proportion of maternal body fat; β: regression coefficient; β': standardized regression coefficient (unit standard deviation); significant codes: p < 0.001 ***, < 0.01 **, ≤ 0.05 *, > 0.05 n.s.

Table S3.b. Fixed and random effects in explaining variation in ln-transformed embryo dry mass in the best linear mixed effect model according to the AICc. The developmental stage of embryos is scaled by subtracting the developmental stage at birth (i.e. stage 45) from the actual developmental stage of the brood. Thus, the maternal main effects (i.e. body fat and standard length) quantify the relationship between the corresponding maternal trait and offspring dry mass at birth. Significance tests for the fixed effects were performed with `lmerTest` (Kuznetsova *et al.* 2016), and confidence intervals for random effects were calculated using `confint.merMod` function implemented in the R package `lme4` (Bates *et al.* 2015).

Fixed effects							
	β	β’	SE	*df*	*t*	*P*	Sig.
Intercept	-0.586	-	0.515	48.974	-1.138	0.261	n.s.
developmental stage[a]	0.008	-	0.001	59.442	5.638	<0.001	***
body fat[b]	2.998	0.279	0.558	74.654	5.369	<0.001	***
standard length	0.020	0.098	0.006	30.025	3.214	0.003	**
body fat[b] × developmental stage[a]	0.011	-	0.003	64.077	3.723	<0.001	***

Random effects			
	Variance	2.5 % confidence level	97.5 % confidence level
mother identity	0.0034	0.0000	0.0727
population identity	0.0000	0.0000	0.1634
residual	0.1783	0.1174	0.2546

[a] scaled developmental stage transformed to the square-root of its third power; [b] arcsin square-root transformed proportion of maternal body fat; β: regression coefficient; β’: standardized regression coefficient (unit standard deviation); significant codes: $p < 0.001$ ***, < 0.01 **, ≤ 0.05 *, > 0.05 n.s.

Table S4.a. Fixed and random effects in explaining variation in ln-transformed embryo lean mass. Fixed effect structure was chosen to be the same as found to best explain variation in embryo dry mass according to the AICc. The maternal main effects (i.e. body fat and standard length) quantify the relationship between the corresponding maternal trait and egg lean mass at fertilization. Significance tests for the fixed effects were performed with `lmerTest` (Kuznetsova *et al.* 2016), and confidence intervals for random effects were calculated using `confint.merMod` function implemented in the R package `lme4` (Bates *et al.* 2015).

Fixed effects							
	β	β'	SE	*df*	*t*	*P*	Sig.
Intercept	-3.128	-	0.428	27.603	-7.309	<0.001	***
developmental stage[a]	0.008	-	0.001	59.460	5.776	<0.001	***
body fat[b]	0.035	0.003	0.535	54.965	0.066	0.948	n.s.
standard length	0.020	0.108	0.006	30.608	3.379	0.002	**
body fat[b] × developmental stage[a]	0.009	-	0.003	63.808	3.229	0.002	**

Random effects			
	Variance	2.5 % confidence level	97.5 % confidence level
mother identity	0.0035	0.0000	0.0663
population identity	0.0000	0.0000	0.1778
residual	0.1655	0.1095	0.2373

[a] developmental stage transformed to the square-root of its third power; [b] arcsin square-root transformed proportion of maternal body fat; β: regression coefficient; β': standardized regression coefficient (unit standard deviation); significant codes: p < 0.001 ***, < 0.01 **, ≤ 0.05 *, > 0.05 n.s.

Table S4.b. Fixed and random effects in explaining variation in ln-transformed embryo lean mass. Fixed effect structure was chosen to be the same as found to best explain variation in embryo dry mass according to the AICc. The developmental stage of embryos is scaled by subtracting the developmental stage at birth (i.e. stage 45) from the actual developmental stage of the brood. Thus, the maternal main effects (i.e. body fat and standard length) quantify the relationship between the corresponding maternal trait and offspring lean mass at birth. Significance tests for the fixed effects were performed with `lmerTest` (Kuznetsova *et al.* 2016), and confidence intervals for random effects were calculated using `confint.merMod` function implemented in the R package `lme4` (Bates *et al.* 2015).

Fixed effects							
	β	β'	SE	*df*	*t*	*P*	Sig.
Intercept	-0.809	-	0.501	49.086	-1.615	0.113	n.s.
developmental stage[a]	0.008	-	0.001	59.460	5.776	<0.001	***
body fat[b]	2.717	0.269	0.538	73.671	5.046	<0.001	***
standard length	0.020	0.108	0.006	30.608	3.379	0.002	**
body fat[b] × developmental stage[a]	0.009	-	0.003	63.808	3.229	0.002	**

Random effects			
	Variance	2.5 % confidence level	97.5 % confidence level
mother identity	0.0035	0.0000	0.0663
population identity	0.0000	0.0000	0.1778
residual	0.1655	0.1095	0.2373

[a] scaled developmental stage transformed to the square-root of its third power; [b] arcsin square-root transformed proportion of maternal body fat; β: regression coefficient; β': standardized regression coefficient (unit standard deviation); significant codes: p < 0.001 ***, < 0.01 **, ≤ 0.05 *, > 0.05 n.s.

Table S5.a. Fixed and random effects in explaining variation in ln-transformed embryo body fat. Fixed effect structure was chosen to be the same as found to best explain variation in embryo dry mass according to the AICc. The maternal main effects (i.e. body fat and standard length) quantify the relationship between the corresponding maternal trait and egg fat at fertilization. Significance tests for the fixed effects were performed with `lmerTest` (Kuznetsova *et al.* 2016), and confidence intervals for random effects were calculated using `confint.merMod` function implemented in the R package `lme4` (Bates *et al.* 2015).

Fixed effects							
	β	β'	SE	*df*	*t*	*P*	Sig.
Intercept	-4.665	-	0.704	29.591	-6.624	<0.001	***
developmental stage[a]	0.011	-	0.002	56.922	5.499	<0.001	***
body fat[b]	0.473	0.034	0.858	56.605	0.551	0.584	n.s.
standard length	0.006	0.023	0.010	32.052	0.591	0.559	n.s.
body fat[b] × developmental stage[a]	0.011	-	0.004	61.845	2.520	0.014	*

Random effects			
	Variance	2.5 % confidence level	97.5 % confidence level
mother identity	0.0362	0.0000	0.1986
population identity	0.0000	0.0000	0.0846
residual	0.3831	0.2531	0.5795

[a] developmental stage transformed to the square-root of its third power; [b] arcsin square-root transformed proportion of maternal body fat; β: regression coefficient; β': standardized regression coefficient (unit standard deviation); significant codes: $p < 0.001$ ***, < 0.01 **, ≤ 0.05 *, > 0.05 n.s.

Table S5.b. Fixed and random effects in explaining variation in ln-transformed embryo body fat. Fixed effect structure was chosen to be the same as found to best explain variation in embryo dry mass according to the AICc. The developmental stage of embryos is scaled by subtracting the developmental stage at birth (i.e. stage 45) from the actual developmental stage of the brood. Thus, the maternal main effects (i.e. body fat and standard length) quantify the relationship between the corresponding maternal trait and offspring body fat at birth. Significance tests for the fixed effects were performed with `lmerTest` (Kuznetsova *et al.* 2016), and confidence intervals for random effects were calculated using `confint.merMod` function implemented in the R package `lme4` (Bates *et al.* 2015).

Fixed effects							
	β	β'	SE	*df*	*t*	*P*	Sig.
Intercept	-1.261	-	0.808	49.777	-1.560	0.125	n.s.
developmental stage[a]	0.011	-	0.002	56.922	5.499	<0.001	***
body fat[b]	3.712	0.268	0.849	73.273	4.373	<0.001	***
standard length	0.006	0.023	0.010	32.052	0.591	0.559	n.s.
body fat[b] × developmental stage[a]	0.011	-	0.004	61.845	2.520	0.014	*

Random effects			
	Variance	2.5 % confidence level	97.5 % confidence level
mother identity	0.0362	0.0000	0.1986
population identity	0.0000	0.0000	0.0846
residual	0.3831	0.2531	0.5795

[a] scaled developmental stage transformed to the square-root of its third power; [b] arcsin square-root transformed proportion of maternal body fat; β: regression coefficient; β': standardized regression coefficient (unit standard deviation); significant codes: p < 0.001 ***, < 0.01 **, ≤ 0.05 *, > 0.05 n.s.

Table S6.a. Fixed and random effects in explaining variation in arcsin square-root transformed proportion of embryo body fat. Fixed effect structure was chosen to be the same as found to best explain variation in embryo dry mass according to the AICc. The maternal main effects (i.e. body fat and standard length) quantify the relationship between the corresponding maternal trait and the proportion of egg fat at fertilization. Significance tests for the fixed effects were performed with `lmerTest` (Kuznetsova *et al.* 2016), and confidence intervals for random effects were calculated using `confint.merMod` function implemented in the R package `lme4` (Bates *et al.* 2015).

Fixed effects							
	β	β'	SE	*df*	*t*	*P*	Sig.
Intercept	0.492	-	0.066	74	7.450	<0.001	***
developmental stage[a]	0.000	-	0.000	74	2.084	0.041	*
body fat[b]	-0.009	-0.012	0.083	74	-0.109	0.914	n.s.
standard length	-0.003	-0.186	0.001	74	-2.849	0.006	**
body fat[b] × developmental stage[a]	0.001	-	0.000	74	1.794	0.077	n.s.

Random effects			
	Variance	2.5 % confidence level	97.5 % confidence level
mother identity	0.0000	0.0000	0.0009
population identity	0.0000	0.0000	0.0027
residual	0.0041	0.0030	0.0058

[a] developmental stage transformed to the square-root of its third power; [b] arcsin square-root transformed proportion of maternal body fat; β: regression coefficient; β': standardized regression coefficient (unit standard deviation); significant codes: p < 0.001 ***, < 0.01 **, ≤ 0.05 *, > 0.05 n.s.

Table S6.b. Fixed and random effects in explaining variation in arcsin square-root transformed proportion of embryo body fat. Fixed effect structure was chosen to be the same as found to best explain variation in embryo dry mass according to the AICc. The developmental stage of embryos is scaled by subtracting the developmental stage at birth (i.e. stage 45) from the actual developmental stage of the brood. Thus, the maternal main effects (i.e. body fat and standard length) quantify the relationship between the corresponding maternal trait and the proportion of offspring body fat at birth. Significance tests for the fixed effects were performed with `lmerTest` (Kuznetsova *et al.* 2016), and confidence intervals for random effects were calculated using `confint.merMod` function implemented in the R package `lme4` (Bates *et al.* 2015).

Fixed effects							
	β	β’	SE	*df*	*t*	*P*	Sig.
Intercept	0.623	-	0.078	74	8.010	<0.001	***
developmental stage[a]	0.000	-	0.000	74	2.084	0.041	*
body fat[b]	0.225	0.296	0.084	74	2.675	0.009	**
standard length	-0.003	-0.186	0.001	74	-2.849	0.006	**
body fat[b] × developmental stage[a]	0.001	-	0.000	74	1.794	0.077	n.s.

Random effects			
	Variance	2.5 % confidence level	97.5 % confidence level
mother identity	0.0000	0.0000	0.0009
population identity	0.0000	0.0000	0.0027
residual	0.0041	0.0030	0.0058

[a] scaled developmental stage transformed to the square-root of its third power; [b] arcsin square-root transformed proportion of maternal body fat; β’: standardized regression coefficient (unit standard deviation); significant codes: p < 0.001 ***, < 0.01 **, ≤ 0.05 *, > 0.05 n.s.

Table S7. Fixed and random effects in explaining variation in maternal fecundity. Fixed effects were chosen to be the same as found to explain variation in embryo dry mass. Significance tests for the fixed effects were performed with `lmerTest` (Kuznetsova *et al.* 2016), and confidence intervals for random effects were calculated using `confint.merMod` function implemented in the R package `lme4` (Bates *et al.* 2015).

Fixed effects						
	β	β’	SE	*z*	*P*	Sig.
Intercept	-1.555	-	0.663	-2.346	0.019	*
body fat[a]	-1.994	-0.034	0.496	-4.022	<0.001	***
standard length	0.067	0.068	0.009	7.606	<0.001	***

Random effects			
	Variance	2.5 % confidence level	97.5 % confidence level
population identity	0.0130	0.0000	0.5285
latest stage	0.0637	0.0000	0.4051

[a] arcsin square-root transformed proportion of maternal body fat; β: regression coefficient; β’: standardized regression coefficient (unit standard deviation); significant codes: p < 0.001 ***, < 0.01 **, ≤ 0.05 *, > 0.05 n.s.

References

Bates, D.M., Mächler, M., Bolker, B.M. & Walker, S.C. (2015) Fitting linear mixed-effects models using lme4. *Journal of Statistical Software*, **67**, 1–48.

Kuznetsova, A., Brockhoff, P.B. & Christensen, R.H.B. (2016) lmerTest: tests for random and fixed effects for linear mixed effect models.
